# Replication and mediation of the association between the metabolome and clinical markers of metabolic health in an adolescent cohort study

**DOI:** 10.1038/s41598-023-30231-9

**Published:** 2023-02-25

**Authors:** Christian Brachem, Leonie Weinhold, Ute Alexy, Matthias Schmid, Kolade Oluwagbemigun, Ute Nöthlings

**Affiliations:** 1 Nutritional Epidemiology, Department of Nutrition and Food Sciences, Rheinische Friedrich-Wilhelms-University Bonn, Meckenheimer Allee 166a, 53115 Bonn, Germany; 2 Institute for Medical Biometry, Informatics and Epidemiology (IMBIE), University Hospital Bonn, 53127 Bonn, Germany

**Keywords:** Lipidomics, Metabolomics, Biomarkers, Biomarkers, Risk factors

## Abstract

Metabolomics-derived metabolites (henceforth metabolites) may mediate the relationship between modifiable risk factors and clinical biomarkers of metabolic health (henceforth clinical biomarkers). We set out to study the associations of metabolites with clinical biomarkers and a potential mediation effect in a population of young adults. First, we conducted a systematic literature review searching for metabolites associated with 11 clinical biomarkers (inflammation markers, glucose, blood pressure or blood lipids). Second, we replicated the identified associations in a study population of n = 218 (88 males and 130 females, average age of 18 years) participants of the DONALD Study. Sex-stratified linear regression models adjusted for age and BMI and corrected for multiple testing were calculated. Third, we investigated our previously reported metabolites associated with anthropometric and dietary factors mediators in sex-stratified causal mediation analysis. For all steps, both urine and blood metabolites were considered. We found 41 metabolites in the literature associated with clinical biomarkers meeting our inclusion criteria. We were able to replicate an inverse association of betaine with CRP in women, between body mass index and C-reactive protein (CRP) and between body fat and leptin. There was no evidence of mediation by lifestyle-related metabolites after correction for multiple testing. We were only able to partially replicate previous findings in our age group and did not find evidence of mediation. The complex interactions between lifestyle factors, the metabolome, and clinical biomarkers warrant further investigation.

## Introduction

Chronic diseases such as cardiovascular disease (CVD), type 2 diabetes mellitus (T2DM), and cancers are among the largest public health burdens modern societies’ face^1,2^. Important for the prevention of these diseases are key modifiable risk factors such as body composition and dietary intake.

Metabolomics is a rich resource in the process of elucidating the etiology of diseases^3,4^. To realize the potential of the metabolome it is important to validate putative biomarkers (henceforth called “metabolites”) and replicate their associations across studies and settings^5^. Well-established clinical biomarkers of metabolic health (henceforth called “clinical biomarkers”), for example cholesterol as a clinical biomarker for CVD^6,7^, HBa1C for T2DM^8^, or inflammation markers (e.g. CRP, IL-8)^4^ appear to be intricately linked with metabolites^3,5,9,10^. However, study findings are largely inconsistent, and might differ by sex and age groups^11–16^ calling for in depth confirmation and replication across sexes and age groups.

Modifiable lifestyle factors including body composition and food intake are linked to a number of chronic diseases such as type 2 diabetes^17–20^, CVD^7,21–23^ or cancer types^24–27^ through alterations in the human metabolome. With respect to prevention, a life course approach elucidates preventive potential in younger age groups, e.g. early adulthood, which has been shown to be relevant^28,29^. In these age groups, clinical biomarkers are of importance to evaluate chronic disease risk. While the relationship of body composition and dietary intake with clinical biomarkers is well reported, less is known on potential mediation through the metabolome. We recently reported associations between body composition and the metabolome (19 metabolites for body mass index (BMI) and 20 for body fat (BF) in urine^30^, as well as between habitual food intake (in food groups) and the metabolome (6 metabolites) in urine and blood^31^). The association of body composition and dietary intake with clinical biomarkers may be linked via some of these metabolites.

To investigate this complex relationship, the aims of the current study were first to identify associations of metabolites with clinical biomarkers based on a systematic literature review (SLR), second to replicate these associations in our study population, and third to evaluate whether our previously reported body composition- and habitual food intake-associated metabolites mediate the association of body composition and habitual food intake with clinical biomarkers. Of note, we focused on the age groups of adolescents and young adults as a particular time window of relevance for prevention.

## Methods

### Systematic literature review

We first conducted a SLR of studies indexed in PubMed, separate for each clinical biomarker, to identify relationships between metabolites and clinical biomarkers to be replicated in our study. A detailed description of the search terms and flow-charts can be found in Additional File S1. Briefly, we included studies that reported associations between Inflammation markers (C-reactive protein (CRP), Interleukin-6 (IL-6), Interleukin-18 (IL-18), Adiponectin, and Leptin), glucose, blood pressure (BP) (systolic blood pressure, diastolic blood pressure, and Hypertension) and blood lipids (high-density lipoprotein (HDL), low-density lipoprotein (LDL), total triglycerides) and the human blood or urine metabolome. We developed a search term for each of these clinical biomarkers. The review was conducted by CB only.

We included all studies where at least one association was reported. We identified additional studies through screening of citations and literature reviews. Information about associations of metabolites and clinical biomarkers was finally extracted from each included study. Of these, only associations reported in at least two independent studies were considered “consistent” and further used in the current study.

### Study design

Both, the confirmation and mediation analyses were conducted in a subpopulation of the DOrtmund Nutritional and Anthropometric Longitudinally Designed (DONALD) study^32,33^. Briefly, the DONALD Study is an ongoing longitudinal open cohort study in Dortmund, Germany, with the goal of analyzing detailed data on diet, growth, development, and metabolism between infancy and adulthood^32,33^. Participants are first examined at the age of 3 months and return for three more visits in the first year of life, two in the second and annually thereafter until the age of 18, when examinations start following a five-year cycle. Examinations include 3-day weighed dietary records (3d-WDR), anthropometric measurements, collection of 24-h urine samples (starting at age 3–4), collection of blood samples (starting at age 18), and interviews on lifestyle and medical examinations. Further details on the study design have been published elsewhere^32,33^.

### Study participants

We included all DONALD study participants that were singletons, full term births (37–42 weeks of gestation) and had a birth weight of at least 2500 g. For the current analysis participants had to have a measurement of both the urine and blood metabolome, as well as at least one measurement of each clinical biomarker. Overall, 218 participants were eligible for the current study.

### Variable assessment

#### Assessment of clinical biomarkers

Inflammation markers (C-reactive protein (CRP), Interleukin-6 (IL-6), Interleukin-18 (IL-18), Adiponectin, and Leptin), glucose, and blood lipids (high-density lipoprotein (HDL), low-density lipoprotein (LDL), total triglycerides) were measured in non-fasted blood plasma. Measurements in blood were always at the same follow-up and from the same sample as metabolome measurement. Blood measurement was always at the same follow-up visit or later than urine metabolome measurement.

Blood pressure (mmHg) was measured multiple times by experienced nursing staff. We used the mean of two repeated measurements for both systolic and diastolic blood pressure. We chose the blood pressure measurement closest after the corresponding metabolome measurement for analysis of the respective participant, which was always at the next study visit.

#### Untargeted metabolomic profiling

The metabolome measurement was already described elsewhere^31^. Briefly, Metabolon Inc. (Morrisville, NC, USA) performed an untargeted metabolomics assay with lipidomics on plasma and an untargeted metabolomics assay on urine samples. For both the plasma and urine untargeted assays, Metabolon used ultra-high performance liquid chromatography-tandem mass spectroscopy (UPLC-MS/MS) to identify metabolites in the samples. Peak identification was done in their propriety Laboratory Information Management System. Compounds were identified by comparison of their retention time/index (RI), mass to charge ratio (m/z) and chromatographic data (e.g. MS/MS spectral data) to library standards. Metabolon maintains a library of authenticated standards with over 3300 commercially available purified standard compounds. Structurally unnamed biochemicals were identified by occurrence. Peaks are quantified using area-under-the-curve and normalized with block correction correcting for inter-day instrument tuning differences. Further details on the metabolic profiling have been reported elsewhere^34^. Both blood and urine untargeted assays were performed in this fashion. Urine metabolite values were additionally normalized to urine osmolality to account for differences in metabolite levels due to differences in the amount of material present in each sample. Metabolon quantified 1042 (811 known and 231 unknown) and 1407 (940 known and 467 unknown) in blood and urine, respectively. A deeper explanation of the metabolomics methods can be found in Additional File S2.

#### Complex lipid platform measurement

Lipids were extracted from samples in methanol:dichloromethane in the presence of internal standards. The extracts were concentrated under nitrogen and reconstituted in 0.25 mL of 10 mM ammonium acetate dichloromethane:methanol (50:50). The extracts were transferred to inserts and placed in vials for infusion-MS analysis, performed on a Shimazdu LC with nano PEEK tubing and the Sciex SelexIon-5500 QTRAP. The samples were analyzed via both positive and negative mode electrospray. The 5500 QTRAP scan was performed in MRM mode with the total of more than 1100 MRMs. Individual lipid species were quantified by taking the peak area ratios of target compounds and their assigned internal standards, then multiplying by the concentration of internal standard added to the sample. Lipid class concentrations were calculated from the sum of all molecular species within a class, and fatty acid compositions were determined by calculating the proportion of each class comprised by individual fatty acids. We identified 966 lipid species in 14 classes as well as 265 fatty acids. A deeper explanation of the lipidomics methods can be found in Additional File S2.

#### Body composition and habitual dietary intake

Body weight and height were measured at every follow-up by experienced nursing staff. Body mass index (BMI) was calculated using height (m) and weight (kg) with the formula $BMI = \frac{{weight}}{{height^{2} }}$. Body fat percent (BF) was calculated from four skin/fold thickness measurements (biceps, triceps, iliaca, and scapula), using age, puberty status, and sex/specific equations from Deurenberg et al.^35^. Previous associations with BMI used in the mediation analysis and further details on body composition assessment were reported in Brachem et al.^30^.

We used multiple annually applied 3d-WDRs to assess habitual food intake on the food group level. Participants had to have at least two 3d-WDR in adolescence (according to the WHO definition, age 10–19). We defined habitual intake as the mean intake across all available records in adolescence. To account for differences in consumed calories, we standardized intake to grams per 1000 kcal. Previous associations with habitual food intake used in the mediation analysis and further details on dietary assessment were reported in Brachem et al.^31^.

### Statistical analysis

Statistical analysis was performed using R software (Version 4.0.3)^36^. All analyses were stratified by sex.

#### Metabolomics data pre-treatment

Both urine and blood metabolite values were log transformed, centered to a mean of zero and scaled to a standard deviation of one prior to analysis.

#### Replication

We used ordinary least squares regression to replicate associations between the metabolites and clinical biomarkers in the DONALD study. The clinical biomarkers were used as the dependent variables and metabolites as the independent variables. We adjusted the models for BMI and age, both at sample collection. Data was split into training (70%) and testing (30%) data to evaluate overfitting. We trained the model on the training data and used these models to predict clinical biomarker values in the test data. Results from the test data were used only to evaluate the model quality. We additionally accounted for multiple testing by holding the false discovery rate at 5%^37^.

#### Mediation analysis

We used causal mediation analysis to evaluate whether our previously reported body composition-^30^ and habitual food intake-related metabolites^31^ mediate the association of body composition and habitual food intake with clinical biomarkers. For the first, BMI and BF were the exposure and the clinical biomarker (BP, IL-6, IL-18, CRP, Adiponectin, leptin, total cholesterol, HDL, LDL, and triglyceride levels) were the outcomes. The 19 (*5-dodecenoylcarnitine (C12:1)*, *7-hydroxyindole* sulfate, *decanoylcarnitine (C10*), *formiminoglutamate*, *glucuronide of C10H18O2* (*12)*, *guanidinosuccinate*, *isobutyrylglycine* (*C4)*, *isovalerylglycine*, *nicotinamide N-oxide*, *proline*, *succinimide*, *thymine*, *tigloylglycine*, *X—12839*, *X—21441*, *X—21851*, *X—24469*, *X—24801*, and *X—25003*) BMI-associated metabolites and 20 (3*-methylcrotonylglycine*, *glucuronide of C10H18O2 (12)*, *glutamine conjugate of C8H12O2 (1)*, *glycine conjugate of C10H14O2 (1)*, *guanidinosuccinate*, *isobutyrylglycine* (*C4)*, *isovalerylglutamine*, *isovalerylglycine*, *nicotinamide N-oxide*, *succinimide*, *tigloylglycine*, *X—11261*, *X—15486*, *X—17676*, *X—21851*, *X—24345*, *X—24350*, *X—24469*, *X—24801*, *X—25442*, and *X—25464*) BF-associated metabolites were considered as mediators. For the second, habitual food intake was the exposure, the aforementioned clinical biomarker markers were the outcomes, and the six (eggs: *indole-3-acetamide*, *N6-methyladenosine*; vegetables: *hippurate*, *citraconate/glutaconate*, *X—12111*; processed and other meat: *vanillylmandelate (VMA)*) food group-associated metabolites were considered as mediators. We used the ‘mediate()’-function in the R package ‘mediation’^38^ for the analysis. We used 1000 simulations (the recommended default) and quasi-Bayesian approximation to estimate the standard errors. We used the model-based approach^38^. The mediator model is the linear regression model that regresses the metabolites on BMI, BF, or habitual food intake adjusted for age at sample collection. The habitual food intake models were additionally adjusted for BMI at sample collection. The outcome model is a linear regression model that regresses clinical biomarker on BMI, BF, or habitual food intake, the mediator (metabolites), and adjustment variables. From these models the causal mediation analysis is performed as described by Imai et al.^39^. Briefly, the model estimates the average causal mediation effect (ACME), which is a numeric measure of how much influence the presence of the mediator has on the total effect of the exposure-outcome association, as well as the average direct effect, the average total effect, and the proportion mediated. We corrected for multiple testing by holding the false discovery rate at 5%.

#### Missing values

We excluded metabolites from the analysis when more than 70% of data was missing. Based on this we excluded 91 and 67 metabolites in female blood and urine, respectively, and 87 and 74 metabolites in male blood and urine, respectively.

For the mediation analysis and the regression models, we performed a single imputation with the “missRanger” package, using 10 trees with a maximum depth of six and three non-missing candidate values for predictive mean matching. We used random forest imputation, as it is recommended for imputation of missing metabolomics data^40^.

#### Sensitivity analysis

We performed sensitivity analysis on the choice of the missing data threshold in the imputation approach, repeating the complete study protocol excluding metabolites with more than 30% missing data (instead of 70% in the main analysis). In males we additionally excluded 103 metabolites and 106 metabolites in blood and urine, respectively, while in females we excluded 123 and 108 additional metabolites in blood and urine, respectively.

### Ethics approval and consent to participate

Informed written consent was obtained from parents and from participants themselves on reaching adolescence. The ethics committee of the University of Bonn, Germany (project identification: 098/06) approved the study. We confirm that all methods were performed in accordance with relevant guidelines and in accordance with the Declaration of Helsinki.

## Results

In the SLR, we found metabolites associated with blood pressure and CRP in at least two independent studies (Table 1). Six metabolites (*4-hydroxyhippurate, Androsterone sulfate, Glutamine, Isoleucine, Phenylalanine,* and *Tryptophan)* for blood pressure and four metabolites (*Betaine, Glutamine, Isoleucine,* and *Tryptophan*) for CRP were present in more than two studies. The full metabolite list we identified in at least one study with their corresponding references can be found in Additional File S3.

**Table 1:** Metabolites associated with conventional systemic markers of chronic disease risk in at least two independent observational studies.

Metabolite	Sources
Blood pressure	
4-Hydroxyhippurate	Zheng et al.^41,42^
Androsterone sulfate	Zheng et al.^41,42^
Glutamine	Goïta et al.^43^, Le Wang et al.^44^
Isoleucine	Liu et al.^45^, Le Wang et al.^44^
Phenylalanine	Hao et al.^46^, Wawrzyniak et al.^47^, Goïta et al.^43^, Meyer et al.^48^, Øvrehus et al.^49^
Tryptophan	Liu et al.^45^, Le Wang et al.^44^
CRP	
Betaine	Jutley et al.^50^, Pietzner et al.^51^
Glutamine	Jutley et al.^50^, Pietzner et al.^51^
Isoleucine	Jutley et al.^50^, Oluwagbemigun et al.^52^
Tryptophan	Jutley et al.^50^, Kosek et al.^53^, Oluwagbemigun et al.^52^

According to systematic search in PubMed. Metabolites without a match in our metabolites and those we did not replicated are available in Additional File S3.

In Table 2, we present characteristics of the DONALD study population. Aside from the BMI at blood sampling, there were no differences for basic characteristics between the sexes. Except for diastolic blood pressure at urine collection, IL-6, IL-18, and total blood triglycerides, all clinical biomarkers were significantly different between the sexes though directions differed. Blood pressure (diastolic at blood draw and systolic at both blood draw and urine collection) and blood glucose were higher in males, while CRP, leptin, adiponectin, total cholesterol, HDL, LDL, and triglycerides were higher in females.

**Table 2:** Characteristics and markers of metabolic health of 218 DONALD participants.

	N	Overall, N = 218^1^	Male, N = 88^1^	Female, N = 130^1^	p-value^2^
BMI [kg/m^2^] at blood draw	218	22.30 [20.65, 24.91]	23.25 [21.21, 26.16]	21.89 [20.31, 24.09]	0.005
BMI [kg/m^2^] at urine collection	218	21.88 [19.96, 23.63]	22.06 [20.38, 23.54]	21.85 [19.88, 23.68]	0.4
Age [years] at blood draw	218	18.00 [18.00, 23.00]	18.00 [18.00, 23.00]	18.00 [18.00, 23.75]	0.5
Age [years] at urine collection	218	18.00 [17.00, 18.00]	18.00 [16.00, 18.00]	18.00 [17.00, 18.00]	0.7
Age difference [years] between last dietary record and blood draw	218	1.00 [0.00, 6.75]	1.00 [0.00, 4.00]	1.50 [0.00, 7.00]	0.7
Age difference [years] between last dietary record and urine collection	218	0.00 [0.00, 0.00]	0.00 [0.00, 0.00]	0.00 [0.00, 0.00]	0.4
3-Methylcrotonylglycine	206	0.99 [0.71, 1.25]	1.00 [0.83, 1.25]	0.94 [0.61, 1.24]	0.075
5-Dodecenoylcarnitine (c12:1)	218	0.93 [0.58, 1.38]	1.18 [0.73, 1.66]	0.84 [0.50, 1.26]	0.001
7-Hydroxyindole sulfate	211	0.92 [0.54, 1.62]	1.25 [0.81, 1.97]	0.68 [0.46, 1.11]	< 0.001
Citraconate/glutaconate	218	1.00 [0.63, 1.80]	1.09 [0.72, 2.00]	0.88 [0.57, 1.68]	0.042
Decanoylcarnitine (c10)	217	0.95 [0.64, 1.44]	0.92 [0.64, 1.58]	1.00 [0.64, 1.39]	0.7
Formiminoglutamate	218	0.97 [0.64, 1.33]	1.01 [0.76, 1.35]	0.85 [0.54, 1.31]	0.020
Glucuronide of c10h18o2 (12)	217	1.00 [0.68, 1.58]	1.03 [0.79, 1.44]	0.98 [0.66, 1.65]	0.4
Glutamine conjugate of c8h12o2 (1)	218	0.98 [0.68, 1.54]	0.98 [0.72, 1.45]	0.99 [0.63, 1.58]	0.7
Glycine conjugate of c10h14o2 (1)	218	0.95 [0.59, 1.68]	1.00 [0.68, 1.92]	0.87 [0.59, 1.63]	0.4
Guanidinosuccinate	218	0.96 [0.70, 1.30]	1.10 [0.81, 1.31]	0.83 [0.61, 1.23]	0.005
Hippurate	218	0.99 [0.69, 1.44]	0.92 [0.69, 1.35]	1.07 [0.70, 1.50]	0.3
Indole-3-acetamide	198	0.93 [0.58, 1.87]	1.06 [0.57, 2.03]	0.88 [0.61, 1.78]	0.5
Isobutyrylglycine (c4)	218	0.98 [0.67, 1.31]	1.08 [0.82, 1.33]	0.87 [0.61, 1.28]	0.011
Isovalerylglutamine	218	0.99 [0.66, 1.36]	1.15 [0.96, 1.44]	0.83 [0.56, 1.16]	< 0.001
Isovalerylglycine	218	1.00 [0.69, 1.44]	1.21 [0.82, 1.51]	0.86 [0.62, 1.34]	< 0.001
N6-methyladenosine	215	0.99 [0.71, 1.42]	1.06 [0.77, 1.44]	0.96 [0.69, 1.41]	0.14
Nicotinamide n-oxide	214	1.01 [0.60, 1.59]	1.00 [0.65, 1.50]	1.05 [0.57, 1.78]	0.7
Proline	218	0.99 [0.69, 1.27]	1.09 [0.78, 1.42]	0.90 [0.66, 1.13]	0.004
Succinimide	217	0.98 [0.70, 1.35]	1.14 [0.90, 1.60]	0.86 [0.60, 1.22]	< 0.001
Thymine	217	1.00 [0.75, 1.33]	1.09 [0.82, 1.42]	0.90 [0.64, 1.23]	0.004
Tigloylglycine	218	0.93 [0.71, 1.28]	1.11 [0.83, 1.41]	0.82 [0.68, 1.23]	0.004
Vanillylmandelate (vma)	218	0.99 [0.79, 1.36]	1.11 [0.86, 1.44]	0.93 [0.76, 1.27]	0.007

^1^Median [IQR].

^2^Wilcoxon rank sum test Males VS Females.

Unknown metabolites are not displayed.

We were able to test associations for 41 of the 46 metabolites associated with clinical biomarkers. We were able to replicate metabolite associations for 10 out of the 41 metabolites we found (Table 3), four out of five for CRP and six out of 36 for blood pressure. We discovered six significant associations in female participants, while there were five significant associations in male participants. Across bio specimens, there were more associations present in urine (six) compared to blood (five). One metabolite, phenylalanine, was associated across sexes with systolic blood pressure. Another metabolite, glutamine, was associated with both diastolic and systolic blood pressure. However, the association between glutamine and diastolic blood pressure for male participants was positive, while the association between glutamine and systolic blood pressure for females was inverse. Across sexes and bio specimen more metabolites (six) were associated negatively. When correcting for multiple testing only the association between betaine and CRP in females remained significant. The complete model results, including direct sex comparisons, can be found in Additional File S4. The test set model metrics can be found in Additional File S5.

**Table 3:** Replicated risk-markers-metabolites association for CRP, diastolic blood pressure, and systolic blood pressure.

Metabolite	Sex	Bio specimen	Super pathway	Sub pathway	β	95% CI	p-value	p-value (FDR)
Clinical biomarker: CRP								
* Betaine*	*Female*	*Blood*	*Amino acid*	*Glycine, serine and threonine metabolism*	*− 0.40*	− *0.61 to *− *0.19*	*0.0002*	*0.0220*
Glutamine	Male	Urine	Amino acid	Glutamate metabolism	− 0.39	− 0.63 to − 0.15	0.0022	0.1008
Isoleucine	Male	Urine	Amino acid	Leucine, isoleucine and valine metabolism	− 0.29	− 0.53 to − 0.04	0.0218	0.2060
Tryptophan	Male	Urine	Amino acid	Tryptophan metabolism	− 0.38	− 0.63 to − 0.13	0.0033	0.1137
Clinical biomarker: diastolic blood pressure								
Glutamine	Male	Blood	Amino acid	Glutamate metabolism	0.25	0.02–0.48	0.0337	0.2682
Clinical biomarker: systolic blood pressure								
4-Hydroxyhippurate	Female	Urine	Xenobiotics	Benzoate metabolism	0.28	0.08–0.48	0.0072	0.1374
Androsterone sulfate	Female	Blood	Lipid	Androgenic steroids	− 0.17	− 0.35 to − 0.00	0.0496	0.3076
Glutamine	Female	Blood	Amino Acid	Glutamate metabolism	− 0.24	− 0.41 to − 0.07	0.0064	0.1368
Phenylalanine	Female	Urine	Amino Acid	Phenylalanine metabolism	0.19	0.00–0.38	0.0477	0.3076
	Male	Blood			0.25	0.02–0.48	0.0328	0.2682
Tryptophan	Female	Urine	Amino Acid	Tryptophan metabolism	0.19	0.00–0.38	0.0466	0.3076

Estimates are generated from linear regression. Models were adjusted for age and BMI, both at sample collection. Metabolites were log-transformed prior to analysis. Estimates and 95% CI are on the log scale.

We controlled the false discovery rate (FDR) at 5% to account for multiple testing. Metabolites significant after correction for multiple testing are marked in italics.

We found no metabolite significantly mediating the relationship of either body composition or habitual food intake and clinical biomarkers after correcting for multiple testing (Table 4). However, we observed two significant total effects, both in male urine. One between CRP and BMI, CRP is estimated to increase by 0.5 standard deviations (SD) as BMI increases by one unit (p-Value (FDR) < 0.0001) and one between leptin and BF, leptin is estimated to increase by 0.62 standard deviations as BF increases by 1 unit (p-Value (FDR) = 0.040). The full model results are available in Additional File S6. The test set model metrics can be found in Additional File S5.

**Table 4:** Metabolites mediating the association of body composition and habitual food intake with clinical biomarkers.

Bio specimen	Sex	Clinical biomarker	Mediating metabolite	Total effect			ACME		
				Estimate	95% CI	p-value^1^	Estimate	95% CI	p-value^1^
Exposure: BMI									
Urine	Male	CRP	5-Dodecenoylcarnitine (C12:1)	0.51	0.254–0.747	0.000	− 0.03	− 0.134 to 0.035	0.983
Exposure: body fat (%)									
Urine	Male	Leptin	Glucuronide of C10H18O2 (12)*	0.62	0.203–1.040	0.040	− 0.10	− 0.285 to 0.018	0.983

Estimates and confidence intervals are in standard deviations.

*ACME* average causal mediation effect, *CRP* C-reactive Protein.

^1^p-values are corrected for multiple testing by holding the false discovery rate at 5%.

In our sensitivity analysis on the amount of missing data we observed one additional significant association after correcting for multiple testing, between glutamine and CRP in male urine (p (FDR) = 0.046, ß = − 0.39, − 0.63 to − 0.15). Additionally, we observed one additional significant total effect in the mediation analysis, between BMI and leptin in male urine and no mediators. The full results tables for the sensitivity analysis can be found in Additional File S7.

## Discussion

In the present study, we conducted an SLR identifying 41 metabolite- clinical biomarker associations (36 for BP, 5 for CRP) that were reported in at least two independent studies. Of these 41, we were able to replicate 10 associations, in our own study population one of which was significant after multiple testing correction. Additionally, we found no evidence of a metabolite mediating the association between body composition or habitual food intake and clinical biomarkers.

### Systematic literature review

We identified 41 metabolites associated with clinical biomarker variables in at least two studies. Interestingly, these were distributed only between two clinical biomarker variables: blood pressure and CRP. Most of the metabolites (36 of 41) were associated with blood pressure. The methods applied to investigate the relationships of the metabolome and the clinical biomarker variables were very heterogeneous. They ranged from correlation analysis (e.g.^54^), through regression (e.g.^46^) to advanced machine learning methods like random forests (e.g.^55^) or PLS (partial least squares) variants (e.g.^56^). Additionally, it would be very useful for future SLR to have an easier format to export all study results in an appendix. Because of the large number of associations usually present in metabolomics studies, this would greatly increase the possibility for future studies to build on. Another important observation in the SLR is that only four out of 50 studies^43,52,57,58^ stratified by sex, with two additional studies having cohorts restricted to either males^59^ or females^60^, though many additional studies adjusted for or matched by sex. Given how strong the influence^11–13,15,16,30,31,52^ of sex is on many different aspects of the metabolome, a better and ideally unified strategy to account for these influences in future studies is needed. Most studies included in the SLR were in exclusively adult study populations. Three studies studied children^53,61,62^ and two studies^52,57^ adolescents and young adults. Age is another influential factor in the composition of the metabolome that may need additional adjustment strategies in the long term^63^.

### Replication

We were able to replicate 10 out of the 41 metabolites testable in our study sample. We found more metabolites replicated in females compared to males (six and five, respectively) and only one metabolite, *phenylalanine*, associated with systolic blood pressure across sexes. In this replication analysis, only one association, the negative association between *betaine* measured in the blood of males and CRP remained significant after correction for multiple testing. *Betaine* is an essential osmolyte derived from either diet or by oxidation of choline^64,65^. Insufficiencies of *betaine* have been associated with many chronic diseases, such as metabolic syndrome, T2DM or vascular diseases^65^. Additionally, *betaine* is considered as an anti-oxidant^64^ and fulfills anti-inflammatory functions^66^. The inverse association between *betaine* in male blood and CRP we observed is therefore in line with the literature.

*Phenylalanine* was not significantly associated with systolic blood pressure after correction for multiple testing but it is interesting. It is the only metabolite associated across sexes and indirectly across bio specimen. It’s association with higher blood pressure is in line with previous literature, that reported a strong association with infant pulmonary hypertension^67^ and more generally elevated cardiovascular risk^68^. Furthermore, it was elevated in metabolically unhealthy obese (compared to metabolically healthy obese)^69^. Because it is a precursor to catecholamines an increase in blood pressure even has a known physiological pathway already^64^. More studies are needed to discern the causal order and exact mechanism of phenylalanine on blood pressure.

### Mediation

We did not identify any metabolite as potential mediator of the relationship between either body composition or habitual food intake and clinical biomarkers.

While we did not identify any mediators in our sample, we still believe there will be mediators identified in the future. Mediators are notoriously hard to identify, as their study requires many association tests (which in turn requires a correction for multiple testing), a large study population and large effect sizes. All three of which were limiting factors within our study.

### Sensitivity analysis

We performed sensitivity analysis on the amount of missing data permitted in the metabolites prior to imputation. We excluded over 100 additional metabolites, but the results did not change in meaning. In the replication analysis, as was expected by reducing the number of metabolites and therefore statistical tests, the metabolite closest to significance in the main analysis was statistically significant in the sensitivity analysis. However, the point estimates of the metabolites remained the same. In the mediation analysis, one additional total effect remained significant after correcting for multiple testing but no mediating effects, the same as the main analysis. Therefore, interpretation of the results was not depended on the choice of missingness permitted in the metabolites prior to imputation.

### Future research

Future research should take the sex differences we reported into consideration in their own study design, ideally by stratification, in order to further our understanding of the physiological differences in metabolism between males and females. A study evaluating the metabolites associated with metabolic health markers as mediators to lifestyle factors would be a great continuation of the present study, ideally in a larger cohort. Lastly, metabolomics would greatly benefit from both a more unified data analysis approach as well as a unified measurement approach to better facilitate meta-analysis and ease the burden of replicating results from different studies.

### Strengths and limitations

The present study has some notable strengths. We used results from our own previous studies to investigate mediation and conducted a SLR to facilitate replication of previously reported associations in the literature. We were able to use global measurements of the urine and blood metabolome in the same participants for both analyses in a comparatively (for metabolomics) large study population. Though the number of statistical tests required for metabolomics in relation to the available data in our study is high, therefore sampling power may be a reason for few total associations found. We employed state of the art statistical analysis and machine learning to investigate both the mediation and the replication. However, we acknowledge several limitations to the study. Our participants are Caucasians (Germans), residing in a large city (Dortmund) and surrounding area and are mostly from a high socio-economic background. This may limit the generalizability of our findings. We used non-fasted plasma samples, which increases the variability of inter and intra participant variability of measurements introducing non-differential measurement error. We constructed habitual diet from multiple measurements in adolescence, which increases the time difference between diet measurement and metabolome assessment. This limits results to more long-term markers but increases the effect size needed to detect a signal. Additionally, we cannot rule out residual confounding by either unknown or unmeasured confounders or related factors such as genetics. In our mediation analysis, we had, compared to other mediation analysis, a relatively small sample size. Lastly, we only have one measurement of the metabolome available, so the temporal reproducibility of these findings is unknown.

## Conclusions

In summary, we identified 41 metabolites associated in at least two independent studies with clinical biomarker and replicated ten associations in our own data, only one of which was significant after multiple testing correction. Additionally, there was no metabolite mediating the relationship between body composition or habitual diet and clinical biomarker. The intricate interplay between lifestyle factors, the metabolome, and metabolic health warrants further investigation.

## Supplementary Information

Supplementary Information 1.

Supplementary Information 2.

Supplementary Information 3.

Supplementary Information 4.

Supplementary Information 5.

Supplementary Information 6.

Supplementary Information 7.

Supplementary Information 8.

## Data Availability

The datasets generated and/or analyzed during the current study are not publicly available due data protection concerns for sensitive data but are available on reasonable request and approval of the principal investigator. Requests can be sent to *epi@uni-bonn.de*. All model results are available in the supplement to this article.

## Abbreviations

CVD: Cardio vascular disease
T2DM: Type 2 diabetes mellitus
BMI: Body mass index
BF: Body fat percentage
SLR: Systematic literature search
CRP: C-reactive protein
IL-6: Interleukin 6
IL-18: Interleukin 18
HDL: High density lipoprotein
LDL: Low density lipoprotein
DONALD: Dortmund nutritional and anthropometric longitudinally designed
3d-WDR: Three day weighed dietary record
UPLC-MS/MS: Ultra-high performance liquid chromatography-tandem mass spectroscopy
RI: Retention time/index
m/z: Mass to charge ratio
BP: Blood pressure
ACME: Average causal mediation effect
SD: Standard deviation
PLS: Partial least squares
