# Supplementary material for: Replication and mediation of the association between the metabolome and clinical markers of metabolic health in an adolescent cohort study
Source: Sci Rep. 2023 Feb 25;13:3296. doi: 10.1038/s41598-023-30231-9 (PMC9968318; doi:10.1038/s41598-023-30231-9)
Supplement: Supplementary file 1 — Supplementary Information 1. [file 41598_2023_30231_MOESM1_ESM.docx]

## Additional Files

**Additional File S1**: File Format: PDF-Document (.pdf). Details on the systematic literature search and prisma flow charts. The search terms for the systematic literature search are documented here as well as the prisma flow chart for each risk factor separately.

**Additional File S2:** File Format: Word-Document (.docx). Details on the metabolomics and lipodomics methods.

**Additional File S3**: File Format: Excel Workbook (.xlsx). Full List of Metabolites with references identified in the systematic literature search.

**Additional File S4**: File Format: Excel Workbook (.xlsx). Full Model Results from the confirmation.

**Additional File S5:** File Format: Excel Workbook (.xlsx). Full test set results from the confirmation and mediation analysis.

**Additional File S6:** File Format: Excel Workbook (.xlsx). Full Model Results from the mediation analysis.

**Additional File S7:** File Format: Word Document (.docx). Results from the Sensitivity Analysis
