# Supplementary material for: Replication and mediation of the association between the metabolome and clinical markers of metabolic health in an adolescent cohort study
Source: Sci Rep. 2023 Feb 25;13:3296. doi: 10.1038/s41598-023-30231-9 (PMC9968318; doi:10.1038/s41598-023-30231-9)
Supplement: Supplementary file 2 — Supplementary Information 2. [file 41598_2023_30231_MOESM2_ESM.pdf]

Details on the systematic literature search and prisma flow charts

Literature Search

Search terms for the literature search

Risk Faktor	Search Term
Blood Pressure	("Metabolomics"[Mesh]) AND ("Blood Pressure"[Mesh] OR "Hypotension"[Mesh] OR "Hypertension"[Mesh] OR "diastolic blood pressure" OR "systolic blood pressure") NOT ("Animals"[Mesh] NOT ("Animals"[Mesh] AND "Humans"[Mesh]))
Cholesterol (Total, HDL, LDL)	(("Cholesterol"[Mesh] OR "Cholesterol, LDL"[Mesh] OR "Cholesterol, HDL"[Mesh]) AND ("Metabolomics"[Mesh] NOT ("Gastrointestinal Microbiome"[Mesh] OR Gut))) NOT ("Animals"[Mesh] NOT ("Animals"[Mesh] AND "Humans"[Mesh]))
CRP	("C-Reactive Protein"[Mesh]) AND ("Metabolomics"[Mesh]) NOT ("Animals"[Mesh] NOT ("Animals"[Mesh] AND "Humans"[Mesh]))
IL - 6	"Interleukin-6"[Mesh] AND ("Metabolomics"[Mesh]) NOT ("Animals"[Mesh] NOT ("Animals"[Mesh] AND "Humans"[Mesh]))
IL - 18	"Interleukin-18"[Mesh] AND ("Metabolomics"[Mesh]) NOT ("Animals"[Mesh] NOT ("Animals"[Mesh] AND "Humans"[Mesh]))
Adiponectin	"Adiponectin"[Mesh] AND ("Metabolomics"[Mesh]) NOT ("Animals"[Mesh] NOT ("Animals"[Mesh] AND "Humans"[Mesh]))
Leptin	"Leptin"[Mesh] AND ("Metabolomics"[Mesh]) NOT ("Animals"[Mesh] NOT ("Animals"[Mesh] AND "Humans"[Mesh]))

Searches were performed in pubmed without additional filters.

We searched pubmed with the documented search terms with no additional filters. Other sources included one review and references.

We screened the titles for inclusion, then abstracts, then full texts. When the study was included all reported metabolite associations were extracted to an excel document with a reference to the study.

Flow Charts

Blood Pressure

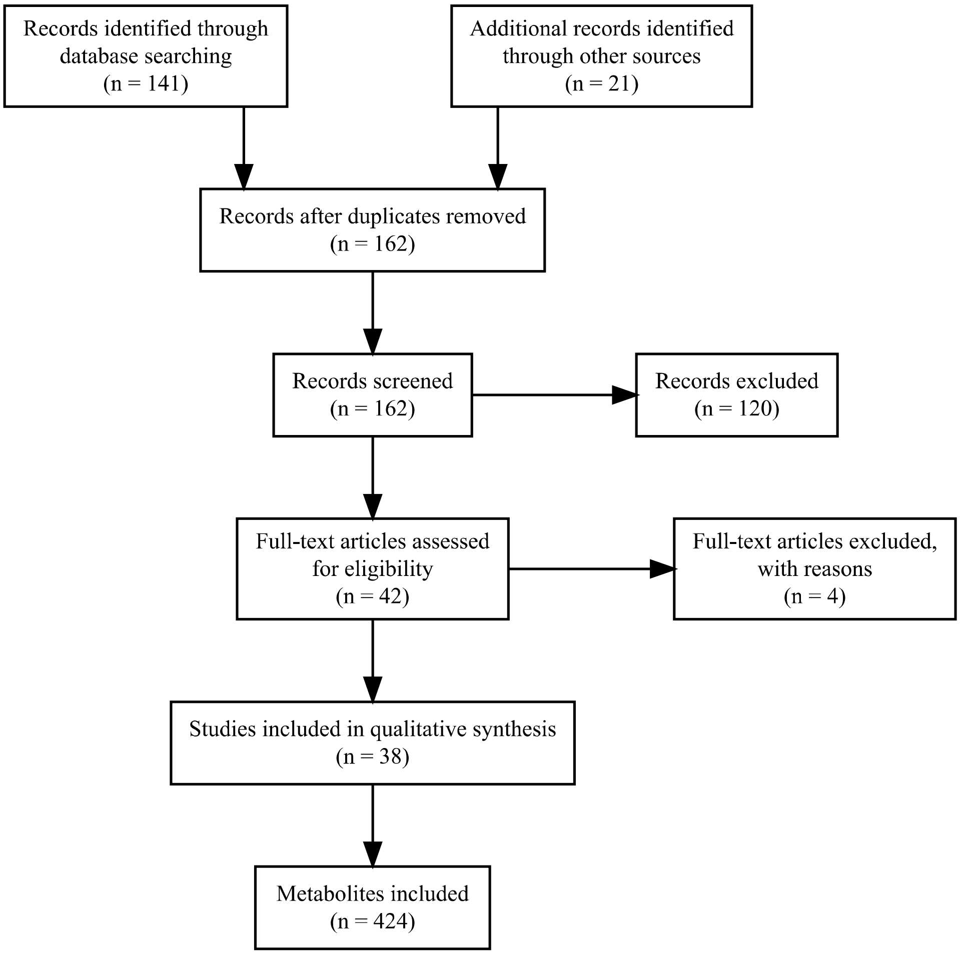

CRP

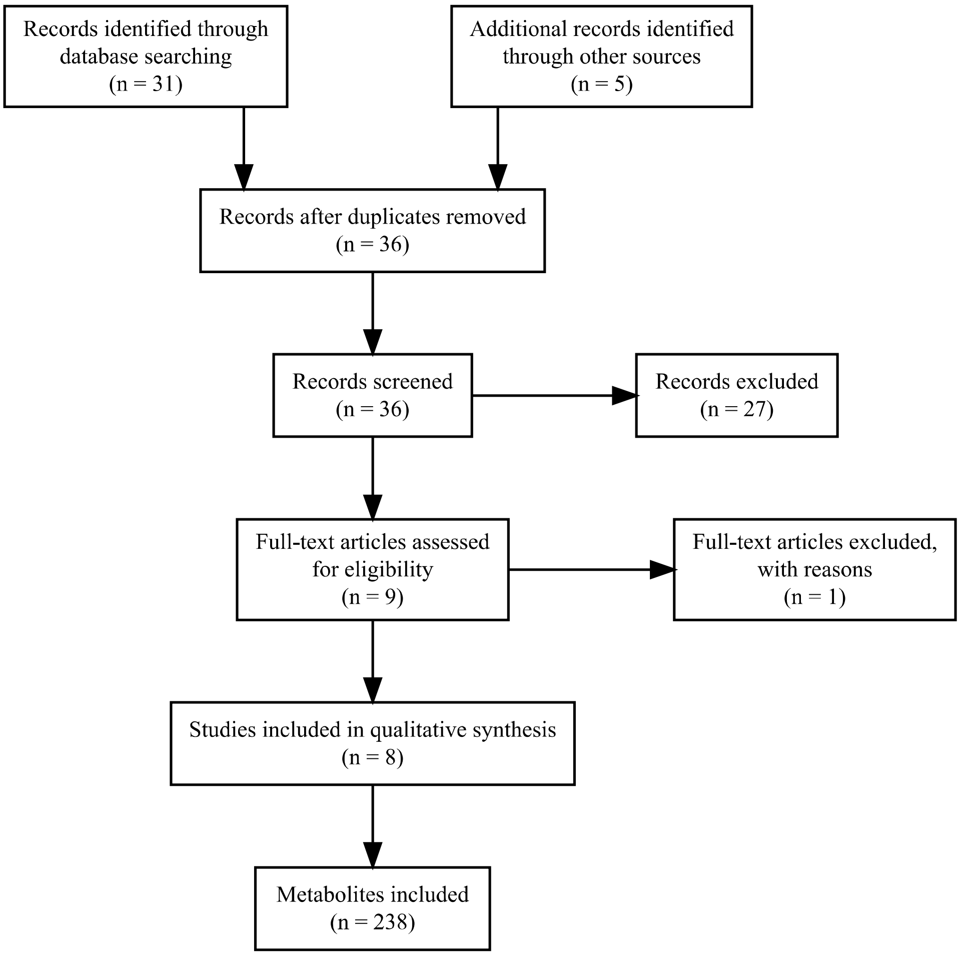
Records identified through database searching (n = 31)
Additional records identified through other sources (n = 5)
Records after duplicates removed (n = 36)
Records screened (n = 36)
Records excluded (n = 27)
Full-text articles assessed for eligibility (n = 9)
Full-text articles excluded, with reasons (n = 1)
Studies included in qualitative synthesis (n = 8)
Metabolites included (n = 238)

IL-6

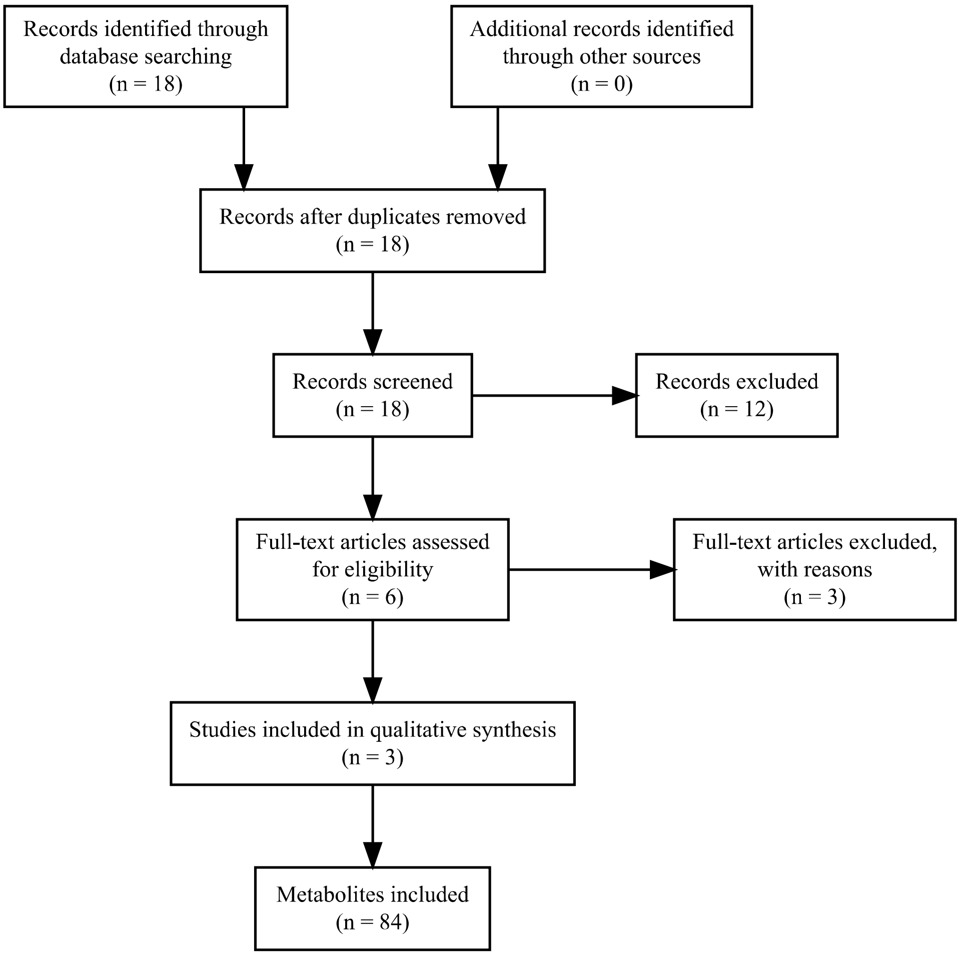
Records identified through database searching (n = 18)
Additional records identified through other sources (n = 0)
Records after duplicates removed (n = 18)
Records screened (n = 18)
Records excluded (n = 12)
Full-text articles assessed for eligibility (n = 6)
Full-text articles excluded, with reasons (n = 3)
Studies included in qualitative synthesis (n = 3)
Metabolites included (n = 84)

IL 18

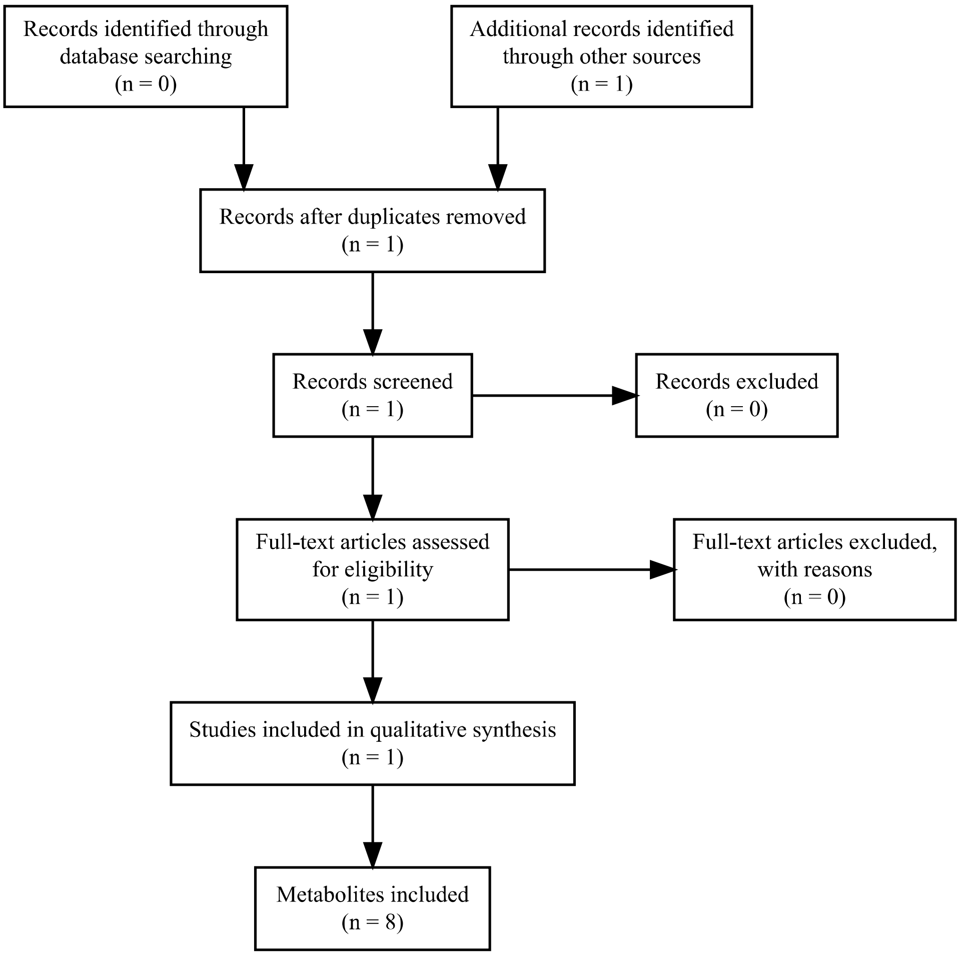

Records identified through database searching (n = 0)
Additional records identified through other sources (n = 1)
Records after duplicates removed (n = 1)
Records screened (n = 1)
Records excluded (n = 0)
Full-text articles assessed for eligibility (n = 1)
Full-text articles excluded, with reasons (n = 0)
Studies included in qualitative synthesis (n = 1)
Metabolites included (n = 8)

Adiponectin

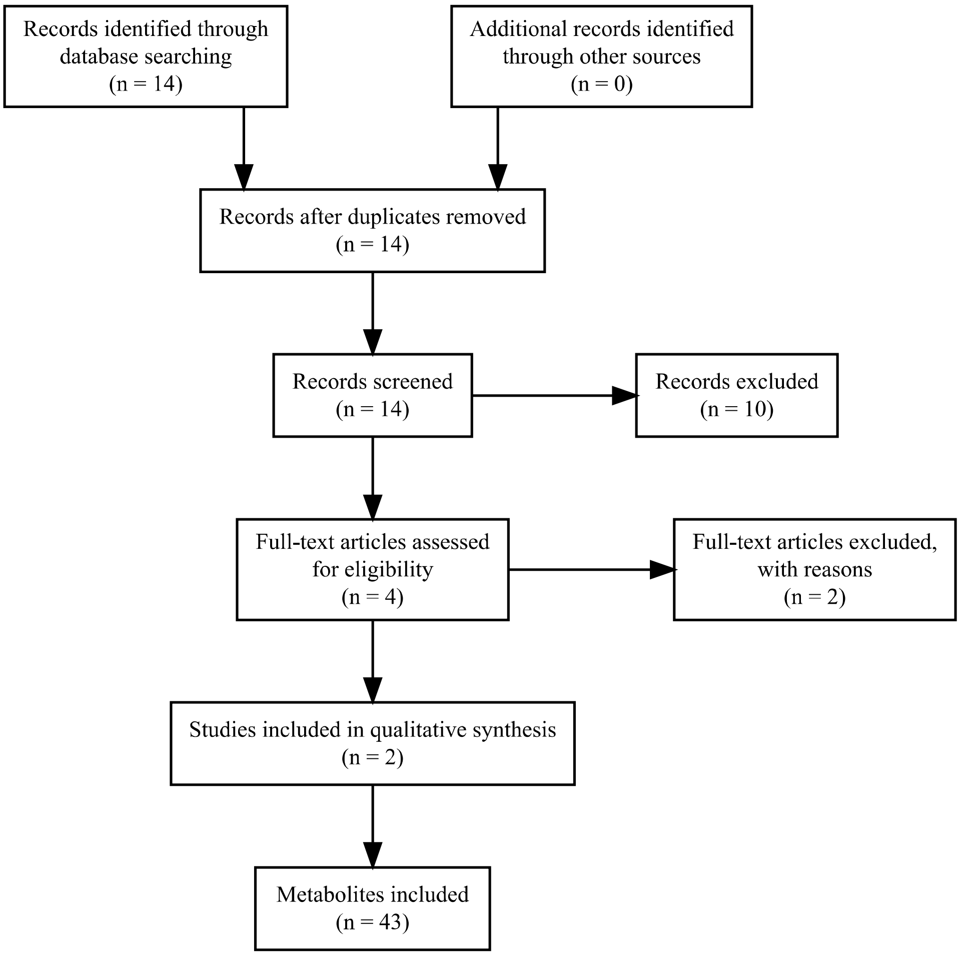

Leptin

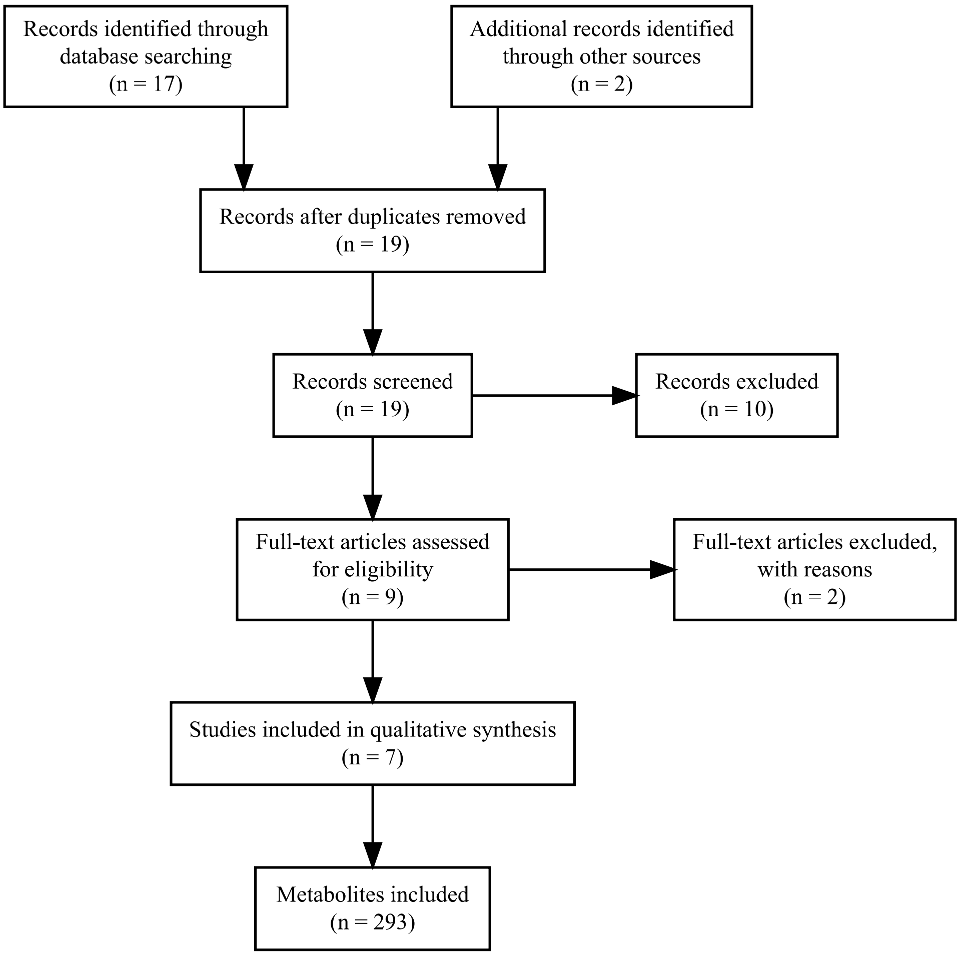

Records identified through database searching (n = 17)
Additional records identified through other sources (n = 2)
Records after duplicates removed (n = 19)
Records screened (n = 19)
Records excluded (n = 10)
Full-text articles assessed for eligibility (n = 9)
Full-text articles excluded, with reasons (n = 2)
Studies included in qualitative synthesis (n = 7)
Metabolites included (n = 293)

Total Cholesterol

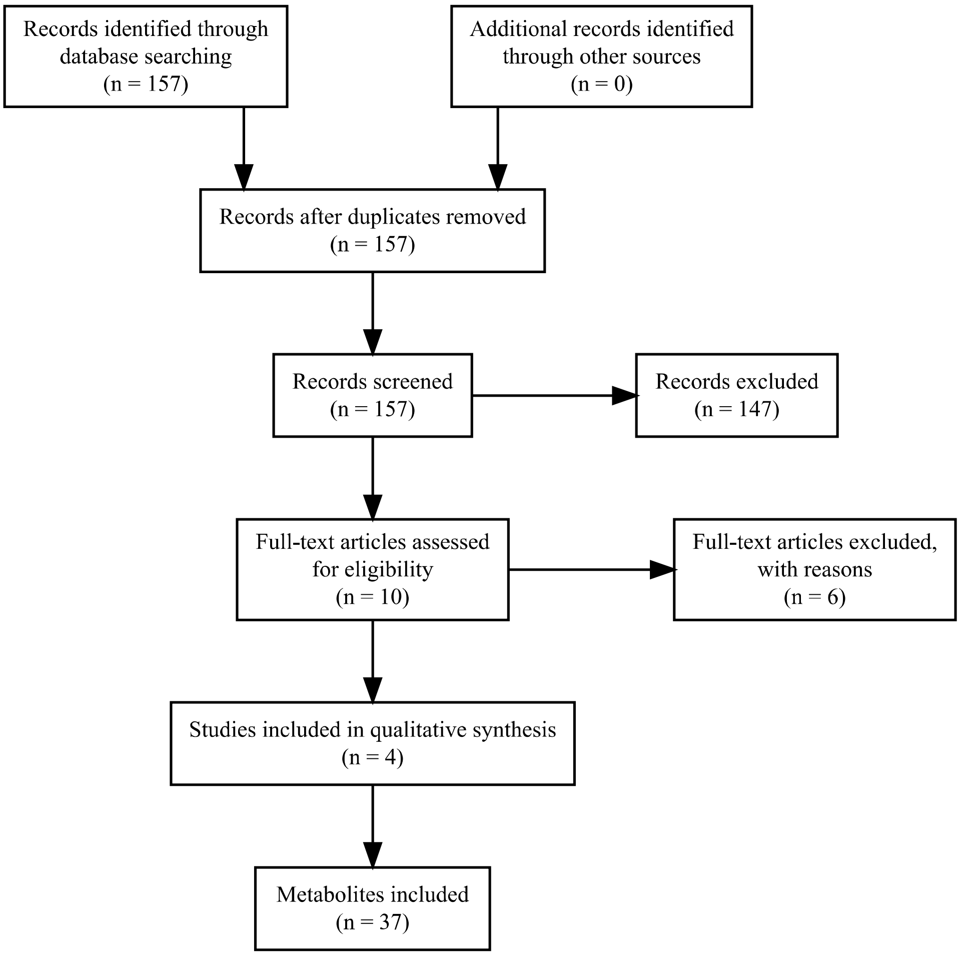

HDL

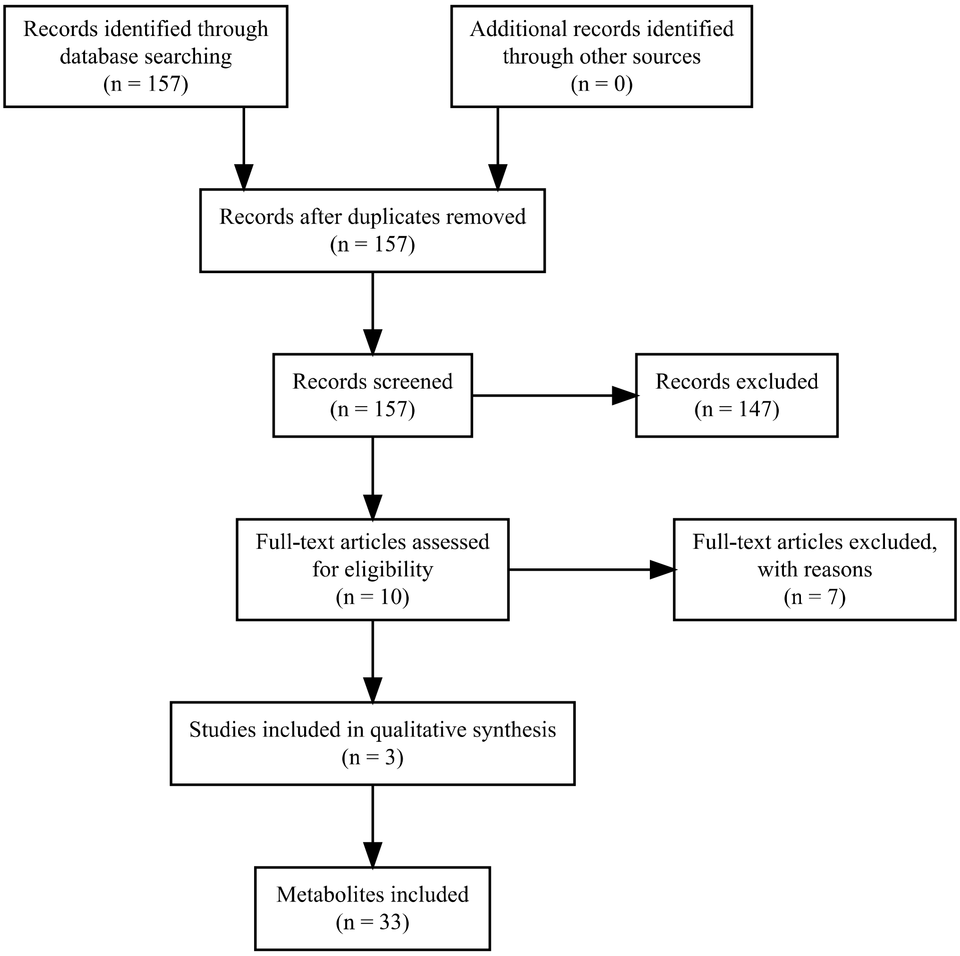
Records identified through database searching (n = 157)
Additional records identified through other sources (n = 0)
Records after duplicates removed (n = 157)
Records screened (n = 157)
Records excluded (n = 147)
Full-text articles assessed for eligibility (n = 10)
Full-text articles excluded, with reasons (n = 7)
Studies included in qualitative synthesis (n = 3)
Metabolites included (n = 33)

LDL

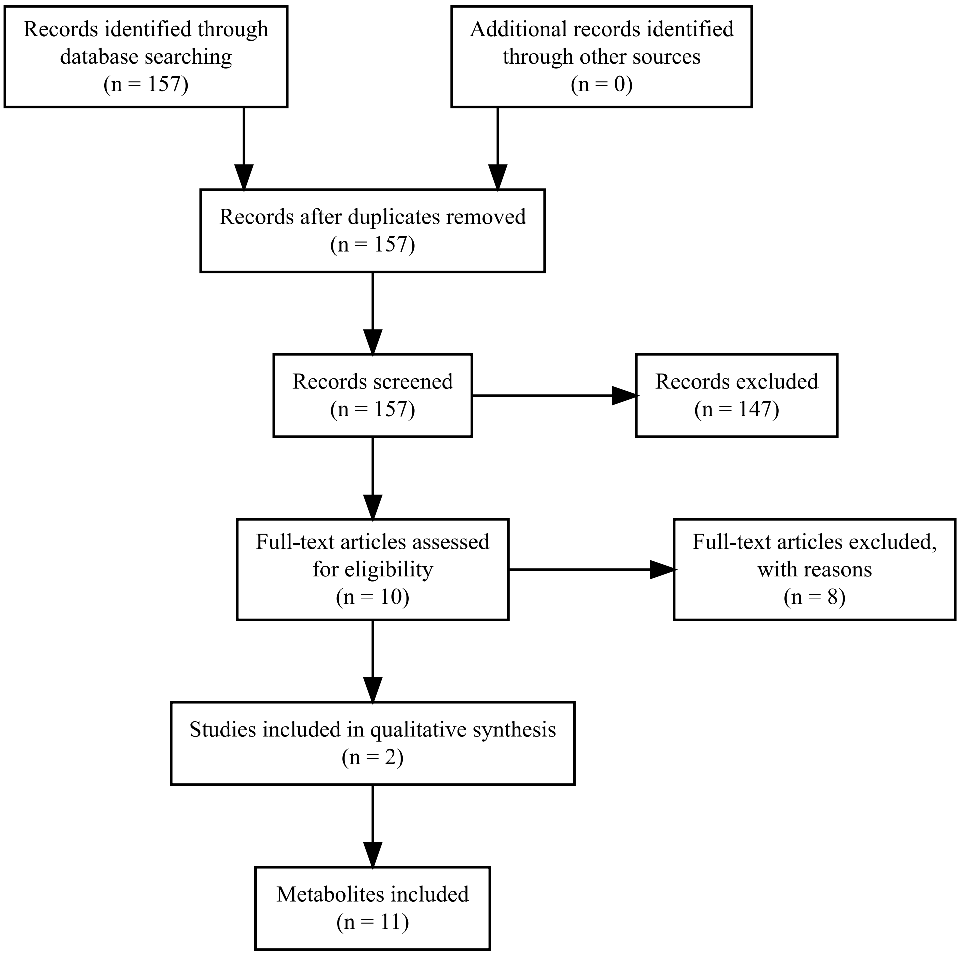
Records identified through database searching (n = 157)
Additional records identified through other sources (n = 0)
Records after duplicates removed (n = 157)
Records screened (n = 157)
Records excluded (n = 147)
Full-text articles assessed for eligibility (n = 10)
Full-text articles excluded, with reasons (n = 8)
Studies included in qualitative synthesis (n = 2)
Metabolites included (n = 11)
