# Supplementary material for: Replication and mediation of the association between the metabolome and clinical markers of metabolic health in an adolescent cohort study
Source: Sci Rep. 2023 Feb 25;13:3296. doi: 10.1038/s41598-023-30231-9 (PMC9968318; doi:10.1038/s41598-023-30231-9)
Supplement: Supplementary file 8 — Supplementary Information 8. [file 41598_2023_30231_MOESM8_ESM.docx]

Table S1: Metabolites replicated from the literature with a 30% missingness threshold

| Metabolite | Sex | Bio specimen | Super Pathway | Sub Pathway | β | 95% CI | p-Value | p-Value (FDR) |
| --- | --- | --- | --- | --- | --- | --- | --- | --- |
| **Outcome: CRP** | | | | | | | | |
| *Betaine* | *female* | *blood* | *Amino Acid* | *Glycine, Serine and Threonine Metabolism* | *-0.40* | *-0.61 to -0.19* | *0.0002* | *0.0066* |
| *Glutamine* | *male* | *urine* | *Amino Acid* | *Glutamate Metabolism* | *-0.39* | *-0.63 to -0.15* | *0.0022* | *0.0462* |
| Isoleucine | male | urine | Amino Acid | Leucine, Isoleucine and Valine Metabolism | -0.29 | -0.53 to -0.04 | 0.0218 | 0.1936 |
| Tryptophan | male | urine | Amino Acid | Tryptophan Metabolism | -0.38 | -0.63 to -0.13 | 0.0033 | 0.0623 |
| **Outcome: Diastolic Blood Pressure** | | | | | | | | |
| 4-hydroxyhippurate | female | urine | Xenobiotics | Benzoate Metabolism | 0.33 | 0.10 to 0.57 | 0.0056 | 0.0810 |
| Glutamine | male | blood | Amino Acid | Glutamate Metabolism | 0.26 | 0.03 to 0.49 | 0.0252 | 0.2144 |
| Phenylalanine | female | urine | Amino Acid | Phenylalanine Metabolism | 0.23 | 0.01 to 0.45 | 0.0389 | 0.2622 |
|  | male | urine |  |  | -0.28 | -0.54 to -0.02 | 0.0347 | 0.2566 |
| Threonine | female | urine | Amino Acid | Glycine, Serine and Threonine Metabolism | 0.22 | 0.01 to 0.43 | 0.0401 | 0.2622 |
| Tyrosine | male | urine | Amino Acid | Tyrosine Metabolism | -0.28 | -0.54 to -0.03 | 0.0280 | 0.2305 |
| **Outcome: Systolic Blood Pressure** | | | | | | | | |
| Glutamine | female | blood | Amino Acid | Glutamate Metabolism | -0.23 | -0.41 to -0.06 | 0.0083 | 0.1087 |
| Phenylalanine | male | urine | Amino Acid | Phenylalanine Metabolism | -0.27 | -0.50 to -0.05 | 0.0194 | 0.1817 |
|  | male | blood |  |  | 0.25 | 0.02 to 0.48 | 0.0337 | 0.2514 |
| Tryptophan | male | urine | Amino Acid | Tryptophan Metabolism | -0.25 | -0.47 to -0.02 | 0.0315 | 0.2407 |
| Tyrosine | male | urine | Amino Acid | Tyrosine Metabolism | -0.25 | -0.47 to -0.03 | 0.0280 | 0.2305 |
| Estimates are generated from linear regression. Models were adjusted for age and BMI, both at sample collection. Metabolites were log-transformed prior to analysis. Estimates and 95% CI are on the log scale.  We controlled the false discovery rate (FDR) at 5% to account for multiple testing. Metabolites significant after correction for multiple testing are marked in bold. | | | | | | | | |

Table S2: Metabolites mediating the association of body composition and food intake with the conventional risk markers of chronic diseases with a 30% missingness threshold

| Bio specimen | Sex | Inflammation Marker | Mediating Metabolite | Total Effect | | | ACME | | |
| --- | --- | --- | --- | --- | --- | --- | --- | --- | --- |
|  |  |  |  | Estimate | 95% CI | p-Value | Estimate | 95% CI | p-Value |
| **Exposure: BMI** | | | | | | | | | |
| urine | male | CRP | 5-dodecenoylcarnitine (C12:1) | 0.51 | 0.254 to 0.747 | 0.000 | -0.03 | -0.134 to 0.035 | 1.000 |
| urine | male | Leptin | isobutyrylglycine (C4) | 0.33 | 0.100 to 0.560 | 0.035 | 0.02 | -0.031 to 0.090 | 1.000 |
| **Exposure: Body Fat (%)** | | | | | | | | | |
| urine | male | Leptin | 3-methylcrotonylglycine | 0.62 | 0.209 to 1.038 | 0.035 | -0.01 | -0.153 to 0.127 | 1.000 |
| Estimates and confidence intervals are in standard deviations.  Abbreviations: ACME- average causal mediation effect, CRP - C-reactive Protein | | | | | | | | | |
